# MR‐linac daily semi‐automated end‐to‐end quality control verification

**DOI:** 10.1002/acm2.13916

**Published:** 2023-02-10

**Authors:** Victor N. Malkov, Jeff D. Winter, Dan Mateescu, Daniel Létourneau

**Affiliations:** ^1^ Radiation Medicine Program Princess Margaret Cancer Center Toronto Ontario Canada; ^2^ Department of Radiation Oncology University of Toronto Toronto Ontario Canada

**Keywords:** adaptive, daily, Elekta Unity, end‐to‐end, MRL, quality control

## Abstract

**Purpose:**

Adaptive radiation therapy (ART) on the integrated Elekta Unity magnetic resonance (MR)‐linac requires routine quality assurance to verify delivery accuracy and system data transfer. In this work, our objective was to develop and validate a novel automated end‐to‐end test suite that verifies data transfer between multiple software platforms and quantifies the performance of multiple machine subcomponents critical to the ART process.

**Methods:**

We designed and implemented a software tool to quantify the MR and megavoltage (MV) isocenter coincidence, treatment couch positioning consistency, isocenter shift accuracy for the adapted plan as well as the MLC and jaw position accuracy following the beam aperture adaptation. Our tool employs a reference treatment plan with a simulated isocenter shift generated on an MR image of a readily available phantom with MR and MV visible fiducials. Execution of the test occurs within the standard adapt‐to‐position (ATP) clinical workflow with MV images collected of the delivered treatment fields. Using descriptive statistics, we quantified uncertainty in couch positioning, isocentre shift as well as the jaw and MLC positions of the adapted fields. We also executed sensitivity measurements to evaluate the detection algorithm's performance.

**Results:**

We report the results of 301 daily testing instances. We demonstrated consistent tracking of the MR‐to‐MV alignment with respect to the established value and to detect small changes on the order of 0.2 mm following machine service events. We found couch position consistency relative to the test baseline value was within 95% CI [–0.31, 0.26 mm]. For phantom shifts that form the basis for the plan adaptation, we found agreement between MV‐image‐detected phantom shift and online image registration, within ± 1.5 mm in all directions with a 95% CI difference of [–1.29, 0.79 mm]. For beam aperture adaptation accuracy, we found differences between the planned and detected jaw positions had a mean value of 0.27 mm and 95% CI of [–0.29, 0.82 mm] and –0.17 mm and 95% CI of [–0.37, 0.05 mm] for the MLC positions. Automated fiducial detected accuracy was within 0.08 ± 0.20 mm of manual localization. Introduced jaw and MLC position errors (1–10 mm) were detected within 0.55 mm (within 1 mm for 15/256 instances for the jaws). Phantom shifts (1.3 or 5 mm in each cardinal direction) from a reference position were detected within 0.26 mm.

**Conclusions:**

We have demonstrated the accuracy and sensitivity of a daily end‐to‐end test suite capable of detecting errors in multiple machine subcomponents including system data transfer. Our test suite evaluates the entire treatment workflow and has captured system communication issues prior to patient treatment. With automated processing and the use of a standard vendor‐provided phantom, it is possible to expand to other Unity sites.

## 1 INTRODUCTION

Through the combination of fast 3D‐imaging sequences and integrated treatment planning workflows, magnetic resonance guided radiation therapy (MRgRT) systems provide a gateway to adaptive radiation therapy (ART).^1^, ^2^, ^3^ On the Elekta Unity, beam apertures are adapted daily based on patient position and anatomy changes. This poses a unique challenge to conventional machine and patient‐specific quality control (PSQC) measurements. On conventional linear accelerators, PSQC measurements can be performed in advance of treatment as verification of plan quality and delivery. Further, the sub‐component performance of the conventional system is tested via the routine quality control (QC) program.^4^ Daily end‐to‐end (E2E) quality control (QC)^5^ is also used on conventional systems to verify image‐guidance radiotherapy (IGRT) performance and workflow.^4^ The Elekta Unity workflow involves a complex interaction of the MR console, record and verify system, treatment planning system (TPS), and the linear accelerator. The complexity of the MRgRT delivery process highlights the need for daily E2E testing on the Elekta Unity parallel to IGRT E2E QC on conventional systems. Such testing ensures the functionality and performance of all system subcomponents to reduce treatment delays caused by otherwise undetected errors.

To address the requirement of an E2E test, Chen et al.^6^ previously developed a daily workflow in which plan adaption is verified through comparison of the isocenter‐shift to a baseline value and visual inspection of MLC positions with respect to a fiducial marker. In our work, we developed a daily E2E test that verifies system transfer components, MR‐to‐MV alignment, daily phantom image registration performance, couch position accuracy, and the overall adapted plan delivery via MLC and jaw position detection. We implemented and streamlined this test at our institution with an in‐house developed Python tool to automate image processing and output high‐quality machine metrics which are sensitive to variations in performance. Our test remains semi‐automated as user interaction is required for phantom setup and transfer of the treatment plan and the MR and MV images. In this work, we present the validation of our E2E test suite through a series of repeatability and sensitivity measurements designed to probe the system response to known introduced errors (MLC, jaw, phantom position, etc.). We further present the clinical results of over 300 test instances of the E2E test suite.

## 2 METHODS

**FIGURE 1:**
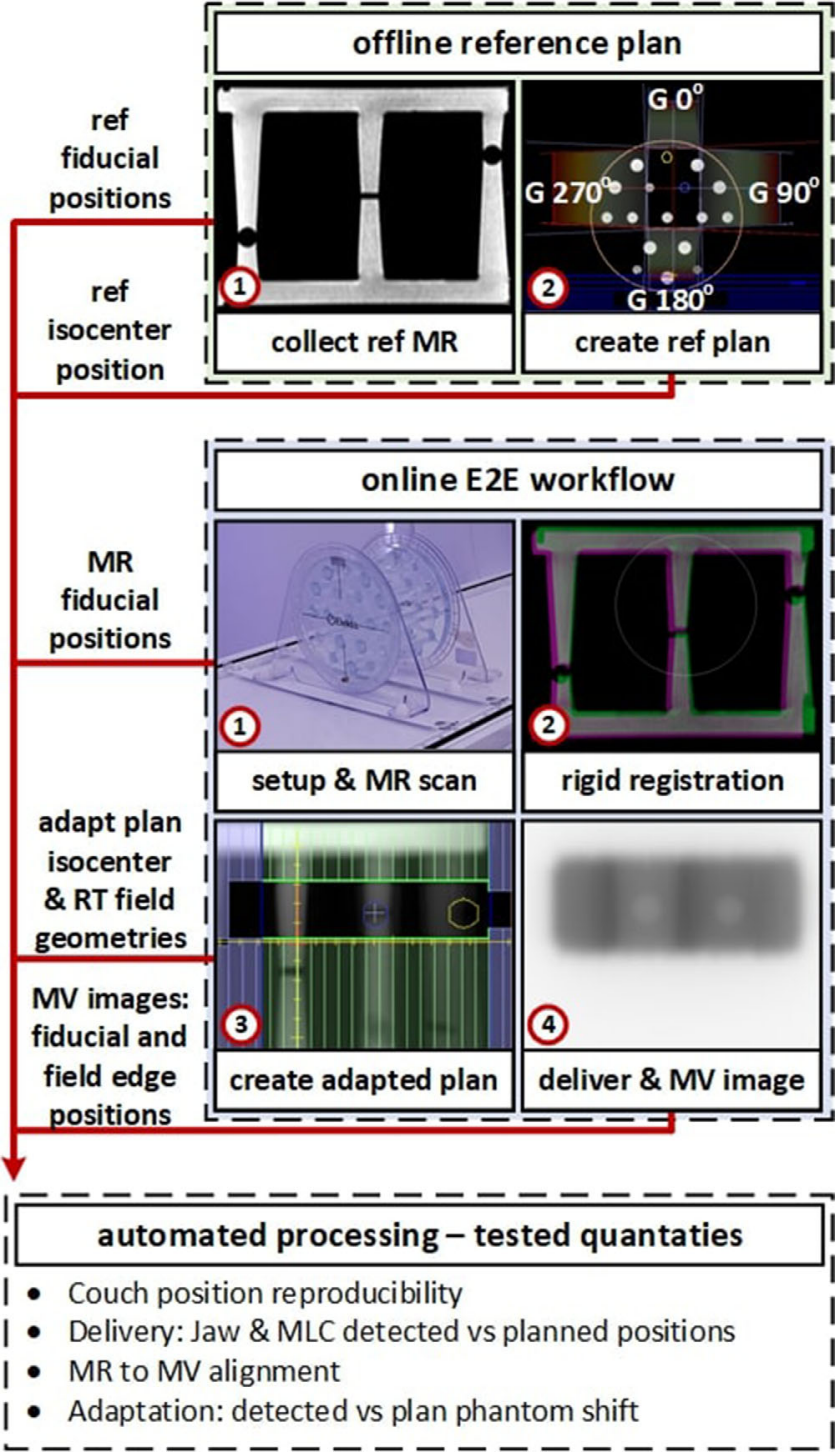
Overview of the E2E workflow. The offline reference plan section depicts the initial preparations steps. Online E2E workflow component is performed at each test instance. Numbered red circles indicate chronological order of steps.

We developed our E2E test suite to quantify the performance of several machine subcomponents. This includes MR‐to‐MV isocenter alignment, couch position consistency, changes to the MV panel isocenter pixel (a gantry angle dependent pixel position on the MV panel which represents the position of the MV isocenter and determined through an independent manufacturer process), the accuracy of the plan isocenter shift (image registration‐based shift), and field delivery accuracy. Our test employs the Elekta MR‐to‐MV phantom (referred to as *phantom*), which contains seven fiducials visible on MR and MV imaging. The overall workflow of our test is highlighted in Figure 1 and Table 1 provide a description of the test components, related processing, and test inputs and outputs.

**TABLE 1:** Subcomponent testing description, required inputs, automation, and test result output

Subcomponent	Description	Input	Software automation	Output
Registration verification	Verify the registration process in Online Monaco (*plan phantom shift)* corresponds to the *detected phantom shift*.	4 MV images Adapted DICOM‐RT plan	Detection of MV fiducials and triangulation of 3D coordinates and comparison with reference plan location: *detected phantom shift* Extraction adapted plan isocenter comparison with reference plan position: *plan phantom shift*.	Difference between the plan and detected phantom shift in x, y, and z directions
Couch position consistency	Verify couch position consistency based on index bar fiducial marker	Gantry angle 0° MV image	Detection of couch fiducial	Fiducial displacement in the x and y direction with respect to baseline
Field delivery verification	Verify delivery of planned fields through detection of the delivered fields.	4 MV images Adapted DICOM‐RT plan	Jaw and MLC field edges detection on MV images Extraction, from the RT plan file, of the planned jaw and MLC positions to determine the planned RT field center	Comparison of planned and delivered jaw and MLC leaf positions for the four fields
MR‐to‐MV alignment	Verify MR‐to‐MV alignment with respect to the reference value	4 MV images Daily MR images	Detection of the 3D position of the fiducials from the MV images Detection of the 3D positions of the fiducials on the MR images.	Registration of MR and MV fiducial markers

### 2.1 Reference plan creation

We collected a phantom reference image with a 6‐min T1‐weighted 3D gradient‐recalled echo (GRE) MR imaging sequence with echo time (TE) = 4.6 ms, repetition time (TR) = 11.0 ms, field‐of‐view (FOV) = 350 mm × 398 mm, slices = 300, SENSE factor = 2.3 acquisition time = 5 min 16 s. Using this reference MR, we designed a treatment plan with four rectangular 3D‐CRT beams to expose the two most anterior fiducials on the superior section of the phantom. We target only two of the fiducials due to the limitations of the MV panel size and the requirement to capture the entire adapted radiation field edges. The plan isocenter is virtually shifted from the center of the phantom in the x and the z directions (IEC 61217; linac coordinate system), whereas a y‐direction (longitudinal) shift is introduced by positioning the phantom offset by an index position from the planned location. From the reference MR and RT plan we extract and store the following in our processing tool: the plan isocenter in the MR image coordinates and the position of the seven fiducials with respect to the isocenter. The fiducials positions on the MR image are extracted using the processing algorithms we established for the daily test.

### 2.2 Daily adaptive workflow

To execute the test, the user places the phantom on the treatment table with the index bar at a predetermined index position. Next, an additional index bar with a small MV visible fiducial is positioned under the phantom to allow for evaluation of the daily couch position. The index bar can be seen Figure 1 under the “setup & MR scan” sub‐figure. The user then collects an MR image of the phantom, performs translation‐only registration of the collected image with the reference plan image, and produces an Adapt‐to‐Position plan (beam segment apertures adapted to account for rigid body translations from the image registration) which is then delivered, and images are captured using the MV imaging panel (pixel size of 0.2163 mm and field‐of‐view of 9.5 cm superior‐inferior and 22 cm left‐right at the isocenter). The image registration results in an isocenter position shift, in the reference MR coordinates, with respect to the reference plan location and we refer to this difference as the *plan phantom shift*. The daily plan adaptation MR has a shorter 2‐min acquisition time to minimize the time required to perform daily QA (TE = 4.6 ms, TR = 11.0 ms, FOV = 400 mm × 400 mm, slices = 300, acquisition time = 1 min 54 s). The user collects and manually exports the MV images of the adapted fields along with the daily MR image and RT plan to the Python‐based automated processing tool.

### 2.3 Evaluated subcomponents

Table 1 provides an overview of the evaluated subcomponents, the associated inputs, processing, and resulting test output. Our tool returns results for couch position consistency, the difference between the planned and detected jaw and MLC positions, MR to MV isocenter alignment, and the difference between the plan and detected a phantom shift. Within the next sections, we provide a more detailed description of the processing and evaluation steps.

### 2.4 Clinical implementation

Here we describe the main steps users would have to take to implement our E2E test at their center. Interested users are encouraged to contact the authors for further information and access to the processing software. To implement our E2E test, users would acquire an MR image of their MR‐to‐MV phantom. The phantom is then imported into Monaco, where a cylindrical external contour encompassing the phantom and an internal target structure would be created. Fiducials in the phantom can be contoured but are not required. The four orthogonal MV fields would be introduced to target the two inferior and anterior fiducials. Importing the plan into Mosaiq, scheduling the treatment, and setting the desired imaging exam card would allow for the generation of adaptive plans.

To initialize the processing software, the phantom must be characterized. This is achieved by gathering 31 MV images, as described in the subsequent sections, with the phantom setup to perform the vendor's MR‐to‐MV alignment QA. Subsequently, the planning MR image, the 31 MV images, and the reference radiation treatment plan is imported into our software. This process sets the reference data for the treatment plan parameters and fiducial positions in the MR‐to‐MV phantom. Further, to initialize the couch position test, users would utilize an index bar with a single high‐density fiducial. The baseline position of the fiducial on the index bar is determined through a set of repeated initial measurements of the daily QA position.

### 2.5 Processing algorithm

In this section, we provide the E2E test suite processing steps. All 3D positions determined in the analysis algorithm are in the IEC 61217 coordinate system. For a head‐first supine patient, the x‐direction is left to right, the y‐direction is inferior to superior, and the z‐direction is posterior to anterior.

#### 2.5.1 Fiducial localization on MV images

We use MV images of the adapted treatment beams to localize the fiducial markers within the phantom. We apply thresholding on each image to isolate the radiation field region. We apply a Gaussian filter (δ = 2) and reduce background contributions within the radiation field by subtracting the average along the *u* and *v* directions. Next, we enhance fiducial edges by applying a Sobel filter and execute the fiducial search in two stages. First, we compute the normalized cross‐correlation (NCC) with a previously generated template at the original image resolution to obtain a rough estimate of marker positions. Next, for each fiducial location, we up‐sample the resolution in the surrounding region by ×10 using tri‐linear interpolation and repeat the NCC, using a high‐resolution template, to obtain the final sub‐pixel fiducial position.

**FIGURE 2:**
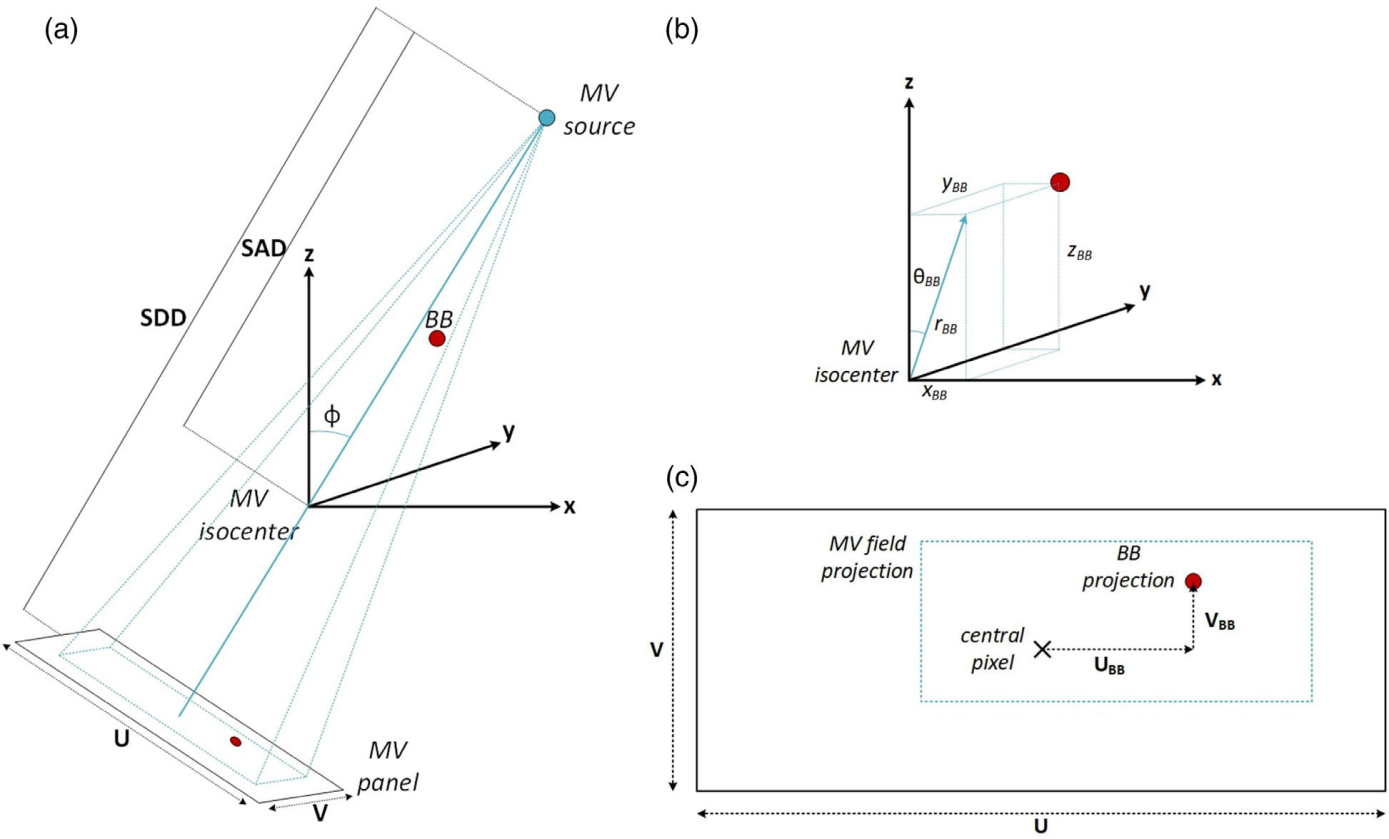
MR‐to‐MV phantom fiducial coordinate system (a) with an illustration of the projection of the fiducial onto the MV panel (b) demonstrating the relevant distances and angle required to localize the fiducial position at each gantry angle. This allows us to determine the projection of the fiducial BB in the u and v coordinates of the MV panel relative to the central pixel of the MV panel.

We extract the 3D position of the fiducials with respect to MV isocenter by triangulating their 2D positions with respect to the *isocenter pixel* on the projected MV images from all gantry angles. The *isocenter pixel* defines the MV panel position which corresponds to the center of a projected fiducial positioned at the isocenter and is set through a manufacturer‐provided process. Gantry angle dependence is included in the *isocenter pixel* value. Figure 2a provides the coordinate system used for describing the position of the fiducial, Figure 2b shows the pertinent parameters describing the source and the MV panel, and Figure 2c provides the coordinate of the fiducial on the MV panel with respect to the isocenter pixel. Based on the geometric information provided in Figure 2A–C, the fiducial positions on the MV panel projected to isocenter are given by:

(1) $$U_{\mathrm{BB}-\mathrm{iso}}\left(\phi \right)=\mathrm{SAD}\left(\frac{\sin\left(\theta_{\mathrm{BB}}-\phi \right)}{\frac{\mathrm{SAD}}{r_{\mathrm{BB}}}-\cos\left(\theta_{\mathrm{BB}}-\phi \right)}\right),$$

and

(2) $$V_{\mathrm{BB}-\mathrm{iso}}\left(\phi \right)=\left(\frac{\mathrm{SAD}}{\mathrm{SAD}-\cos\left(\theta_{\mathrm{BB}}-\phi \right)r_{\mathrm{BB}}}\right)y_{\mathrm{BB}}.$$

**FIGURE 3:**
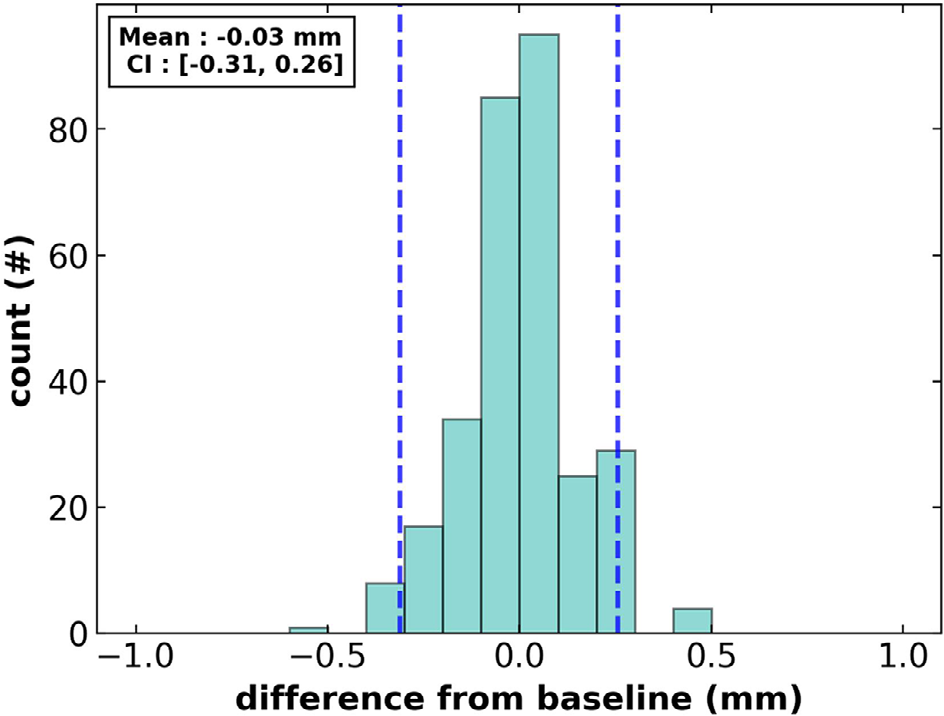
Histogram of couch x‐ray marker distance from the baseline value position. Dashed lines represent the 95th percentiles of the histogram.

The MV pixel index coordinates are converted using $U_{BB-iso}=\;U_{BB}*PR$ and $V_{BB-iso}=\;V_{BB}*PR$, where PR is the pixel resolution (0.2163 mm/pixel). The fiducial positions on the MV panel as a function of ϕ can be plotted and subsequently Equations 1 and 2 can be fit to determine $\theta_{BB},\;\;r_{BB}$, and $y_{BB}$. The $\theta_{BB}$ and $r_{BB}$ are then transformed into the corresponding $x_{BB},\;y_{BB}$ and $z_{BB}$.

#### 2.5.2 Fiducial localization on MR image

The fiducials show up as a signal void on the MR images and we localize these in the MR coordinate system for each test run. We determine the fiducial position through a three‐stage process that aims to obtain sub‐pixel accuracy. First, we determine the inferior‐left‐posterior edge of the phantom in the image by a threshold search of a flattened image. Based on this position and the previously determined phantom characteristics, we extract a 3D section around the rough position of each fiducial. This method allows us to reduce the computational burden by limiting the search volume. We average the 3D volume in either the x or z directions to generate two 2D images on which we apply a Sobel filter and perform two 2D NCC computations with pre‐computed templates to find a more accurate fiducial position based on the maximum correlation points. Based on this refined position, we extract two slices from the 3D image along the x and z directions. On each of these slices, we apply a Sobel filter and spline‐interpolation up‐sampling (×20). This process accentuates the edges of the fiducials which appear as two bright half circles (one superior and one inferior). We determine the position of the bright edges of the half circles along a sampling of 150 profiles and perform a quadratic fit of these points to report the parabola center as the final sub‐pixel fiducial position on the 2D slices. We use these 2D points to report the corresponding 3D position in IEC 61217 coordinates.

#### 2.5.3 Radiation field—Jaw and MLC detection

To determine the field edge positions, we estimate the field center by image thresholding and corner detection. Based on the central pixel, for each MLC leaf within the radiation field, we extract 11 adjacent profiles along the *u* direction and determine the superior and inferior MLC leaf edges on the profiles. We average these values to produce the final superior and inferior MLC edges for that leaf pair. The *u* position of the MLC leaves (leaf centers) is determined by using the isocenter pixel and known MLC width (7.15 mm). The corresponding width of the 11 adjacent profiles is 2.38 mm. For this detection mechanism to fail, the isocenter pixel would have to be offset from the true isocenter by 2.38 mm in the *u*‐direction, which would indicate a significant MV panel shift or a change in the MV isocenter. To determine the jaw field edge, we extract 11 adjacent profiles in the v‐direction from the coarse radiation field center and the average result of the profiles provides the detected jaw positions. For all analyzed profiles (under the MLC leaves or the jaws), we apply a Gaussian filter (δ = 3), numerically calculate the gradient, up‐sample the resolution by 20× via spline interpolation and determine the edge position based on the maximum gradient location. By using a gradient‐based method we increase the robustness of the field edge detection against background variations (such as the presence of the phantom in the MV images).

#### 2.5.4 Couch fiducial detection

To characterize the couch position consistency, we included a CT scanner skin marker on an index bar placed underneath the phantom. To detect this marker, we interrogate the MV image collected at gantry 0⁰ by cropping a pre‐determined region. We remove the background from this region by evaluating the average pixel value in the *u* and *v* directions, up‐sample the image (×10) via bi‐linear interpolation and extract the marker position based on the maximum intensity pixel.

#### 2.5.5 Phantom geometric characterization

We characterize the position of the seven zirconium fiducials in the phantom to account for manufacturing variations. Three of the fiducials, distributed on an equilateral triangle, are positioned on an inferior plane of the phantom, and the remaining four, also distributed on an equilateral triangle, are positioned on a superior plane. We collected 31 MV images of the phantom from different gantry angles (spaced 3° apart and avoiding high attenuation regions) which is identical to the Elekta set‐to‐work procedure used to determine the MR‐to‐MV alignment. We track each of the fiducials as a function of the gantry angle and apply the triangulation procedure described in Section 2.5.1 to determine the fiducial positions with respect to the average position of all the fiducials,  ${\vec{x}}_{avg-ref}$. We store the distance of each of the fiducials with respect to ${\vec{x}}_{avg-ref}$ and the corner of the phantom. We use these quantities in evaluating the MR spatial geometry and accelerating fiducials localization on MR images.

#### 2.5.6 MR‐to‐MV alignment evaluation

The MR‐to‐MV alignment describes the physical differences between the MR isocenter and the MV radiation isocenter. In the E2E test suite, we obtain the 3D coordinates of two superior fiducials using the MV images and the corresponding fiducial positions in the MR images. We then register the MR and MV coordinate fiducials by minimizing the root‐mean‐square sum of the difference of the fiducial positions, under an applied rigid shift of the MV positions. The determined shift represents the MR‐to‐MV isocenter alignment.

### 2.6 Evaluation of test sensitivity

We performed a series of tests to evaluate the E2E test suite's sensitivity to errors and uncertainties. This has allowed us to characterize and validate test performance for a variety of parameters. We provide further test details in the supporting documentation. We evaluated the E2E test suites’ performance with the following: (i) reproducibility (variation given the same test conditions) via repeat measurements with the same phantom position; (ii) test repeatability (variation under new test conditions) and impact of applying the adaptive process by performing repeat measurements with new phantom setup at each measurement and collecting both the original reference segments and the adapt‐to‐position segments; (iii) beam aperture position detection by introducing known offsets in the jaw and MLC positions; (iv) impact of the presence of the phantom on the MLC detection by performing sequential imaging with and without the phantom for the same radiation fields; (v) MLC physical positioning and detection repeatability by evaluating repeat MLC detection for static or re‐setup MLC positions; and (vi) detection of known introduced phantom shifts from a reference position.

### 2.7 Clinical implementation and evaluation

We introduced the E2E test as a daily QC test for our Unity system prior to the first patient treatment. Initially, the test was implemented using the CT‐based reference plan, but we switched to an updated version of the test using an MR‐based reference plan. The switch was required as the CT‐to‐MR image registration of the rigid phantom contributed significant uncertainty in the comparison of the planned and measured phantom shifts and matched our change clinically from CT‐ to MR‐based reference plans. Multiple physics associates performed the test each morning, as such results include test runs performed by multiple users.

#### 2.7.1 Overall evaluated quantities

For each test instance, the MV images, MR images, and the adapted DICOM‐RT are exported and loaded into the software. The software extracts the adapted isocenter position (in the image coordinates—the physical machine isocenter is fixed) and the planned RT fields (angle, jaw positions, and individual MLC numbers and positions) from the adapted DICOM RT plan file. We capture the reference isocenter position from the reference plan DICOM RT plan, which is used for all subsequent analyses. We determine the 3D coordinates of the two irradiated fiducials on the MV images and the 3D coordinates of seven fiducials on the MR images. From the MV images, we also extract the detected MLC and jaw positions and the location of the couch fiducial. The resulting tested quantities are described in Table 2. Further, Table 2 provides a brief justification for the inclusion of individual subcomponent testing. Compared with previous implementations, our test suite provides a quantitative evaluation of the adaptive and delivery process while leveraging the acquired data to extract additional information such as the MR‐to‐MV alignment.

**TABLE 2:** E2E test suite evaluated test quantities and relationship to parameters determined through automated processing

Tested quantity	Evaluation method	Inclusion justification	Set tolerance
Registration/ adaptation verification	Difference between the plan phantom shift and the detected phantom shift	Fusion performance and application of MR‐to‐MV alignment in Online Monaco	±1.5 mm in each x, y, and z directions
Couch position consistency	Difference of detected couch fiducial position and baseline value	Ongoing tracking of couch position performance and confirmation patient motion	±0.5 mm
Delivery verification	Difference between detected and planned jaw and MLC positions	Provides verification of the file transfer components between Online Monaco, Mosaiq, and the linac. Jaws and MLC performance tracking	±1 mm
MR‐to‐MV alignment	Difference between determined MR‐to‐MV alignment and set alignment in the Unity system	Independent verification of the MR‐to‐MV alignment	±0.5 mm in each x, y, and z directions

## 3 RESULTS

Over our daily testing, we have collected a total of 86 and 301 instances of the test with the reference plan generated on a CT and MR of the phantom, respectively. We found the CT‐based results had increased variability of the automated image registration in Online Monaco due to different features being present in the phantom in both modalities. This variation was predominantly in the y‐direction and resulted in multiple instances of more than 1.5 mm disagreement between the detected phantom shift and the online Monaco registration. We present the results from the MR‐based E2E test suite for all aggregate daily analysis except for the MR‐to‐MV results which are not impacted by the choice of the reference imaging modality.

### 3.1 Validation of fiducial localization

We determined the accuracy of the fiducial marker detection on MV images using 131 (29 images) manually localized fiducials. We found the average deviation (± one standard deviation) between the automated and manually identified fiducial locations were –0.04 ± 0.08 mm in the *u* direction and –0.02 ± 0.11 mm in the *v* direction. We perform a similar comparison for the MR localization (n = 70, 10 images) and found that the difference between individual fiducial localization using manual and automated methods is –0.03 ± 0.07 mm, –0.05 ± 0.17 mm, –0.08 ± 0.20 mm in the x, y, and z directions, respectively.

### 3.2 Evaluation of test sensitivity

In the supplemental material, we provide detailed results of the sensitivity testing of the E2E test suite. Bracketed terms in the results represent the 95% confidence intervals.

- Evaluation of test result reproducibility through 20 measurements with the couch in the same positions produced: couch fiducial position within 0.28 mm from the measurement mean (95% CI), MR‐to‐MV alignment of –0.02 [–0.08, –0.03] mm (x), –0.50 [–0.54, –0.47] mm (y), and 0.33 [0.28, 0.40] (z), the difference between the planned and detected phantom shift with the largest 95% confidence interval magnitude of 0.52 mm, and the maximum 95% CI of the difference between the plan and detected MLC and jaw was 0.78 mm.
- Evaluation of plan adaptation through test‐retest with 20 measurements of each with and without adaptation with the phantom setup a total of 20 times showed a difference between the plan and detected phantom shift was consistent with the nominal shift introduced in the E2E test when the original plan was delivered (no image registration performed in Online Monaco). This process demonstrated the application of the adaptation within Monaco and allowed for straightforward process of verification.
- Field edge detection accuracy was determined through the introduction of known offsets of 1 to 10 mm for MLC and jaw positions from a reference field. All MLC offsets were detected to be within 0.55 mm and jaw offsets were within 0.5 mm of the expected positions for 250 of 265 measurements and within 1 mm for the remaining 15 measurements.
- Assessment of MR‐to‐MV phantom on field edge detection through irradiations (n = 20) with and without the phantom. The maximum average difference between the two cases was 0.14 ± 0.13 mm for the lower (Y1) MLC leaves at a gantry angle of 270°.
- Evaluation of MLC leaf setup variation through 20 repeated field deliveries, without the phantom present, showed average MLC position difference (Figure 1A ‐ supplemental) of 0 ± 0.02 mm if not resetting up the field between deliveries and 0 ± 0.07 mm (1σ) if resetting up fields (deviations beyond 0.1 mm notably increased).
- Assessment of phantom position detection accuracy via MR and MV through introduced 1, 3, and 5 mm shifts of the phantom from a reference position. A worst‐case standard deviation of 0.26 mm was determined across all the shifts for both imaging modalities.

### 3.3 Clinical implementation results

The clinical results we present in this section are from a total of 301 runs of the E2E test suite on our Elekta Unity MRL. These results represent over a year's worth of machine performance tracking. Each of the tests runs taken roughly 10–15 min to complete. As seen in Figure 3, the couch fiducial position consistency analysis produced a mean –0.03 mm [–0.31 mm, 0.26 mm] (95% CI). To set the tolerance for all future tests, we calculated the maximum deviation for the first 25 E2E measurements acquired clinically, resulting in a ±0.5 mm on the couch consistency tolerance.

**FIGURE 4:**
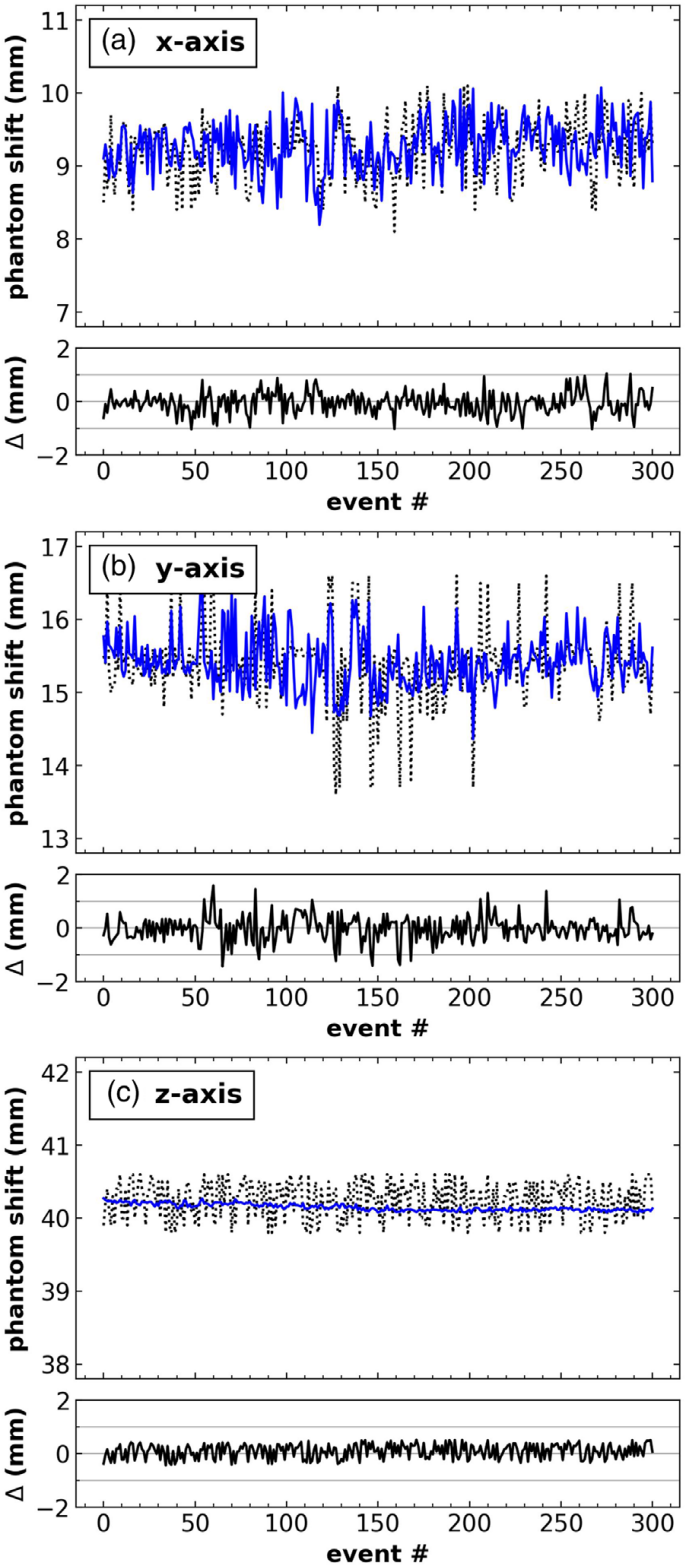
Test instance results of the detected and plan phantom shift in the (a) x, (b) y, and (c) z directions. Solid blue lines in the upper sections represent the detected phantom shift, dashed lines are the plan phantom shift, and the lower section is the difference between the two.

In Figure 4 we plot the values and differences between the detected phantom shift (MV image‐based) and the plan phantom shift (Monaco registration based). The aggregate analysis produces a mean and 95% CI of the difference between the two values of 0.05 mm [–0.84, 0.87 mm] in X, 0.02 mm [–1.04, 0.98 mm] in Y, and –0.10 mm [–0.49, 0.36 mm] in Z. Greatest differences between the detected and planned isocenter shift existed in the y‐direction due to the uncertainty of the automatic rigid registration in Online Monaco which is likely caused by the distinct flat phantom edges in this direction. The associated tolerance level set for this test is ±1.5 mm.

**FIGURE 5:**
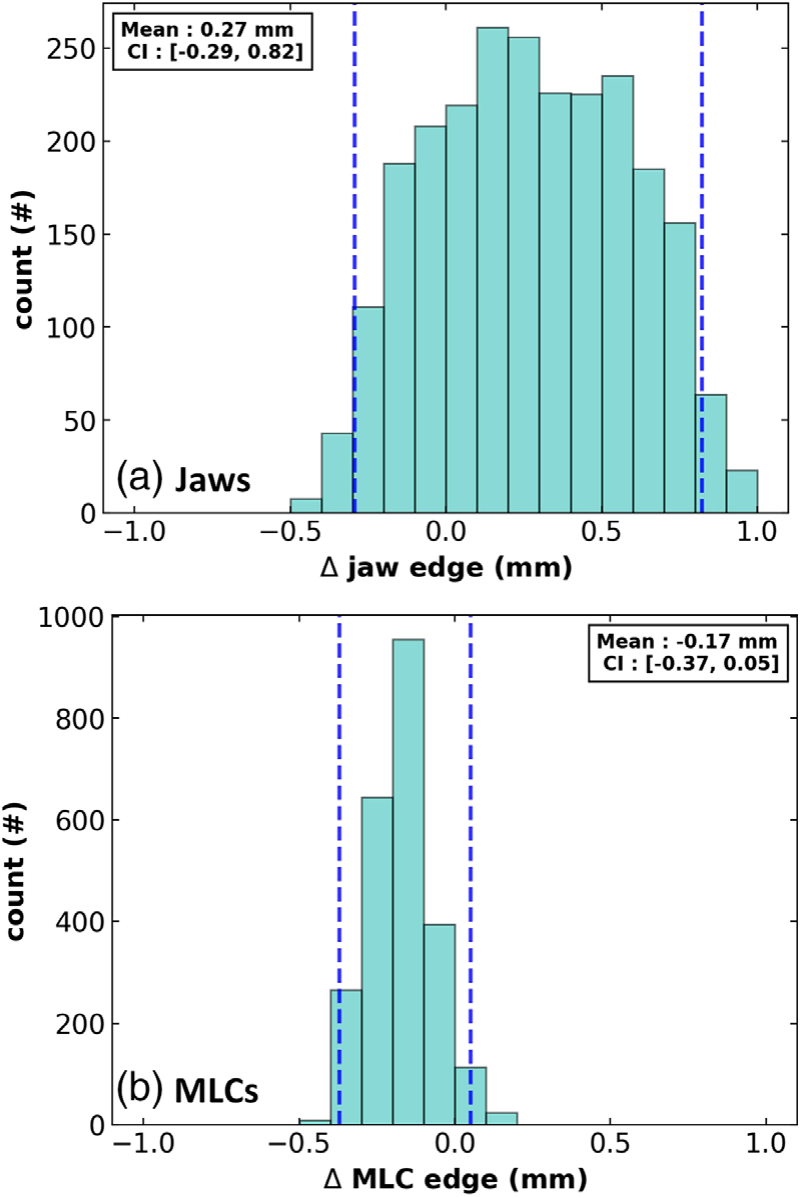
Histogram of the differences in radiation field centers between the planned position and that determined on the MV image in the (a) jaw and (b) MLC directions. Dashed lines represent the 95th percentiles for each histogram.

In Figure 5, we plot the histograms of the difference between the planned and detected radiation field edges defined by the MLCs and jaws. Data from all four gantry angles are combined in these plots. The mean and 95% CI are 0.27 mm [–0.29, 0.82 mm] in the jaw direction and –0.17 mm [–0.37, –0.05 mm] in the MLC direction. In Figure 3A (supplemental) of the supporting documentation, we demonstrate our tool's capability to track individual MLC performance. The tolerance level for the MLC and jaw average position is set at ±1.0 mm.

**FIGURE 6:**
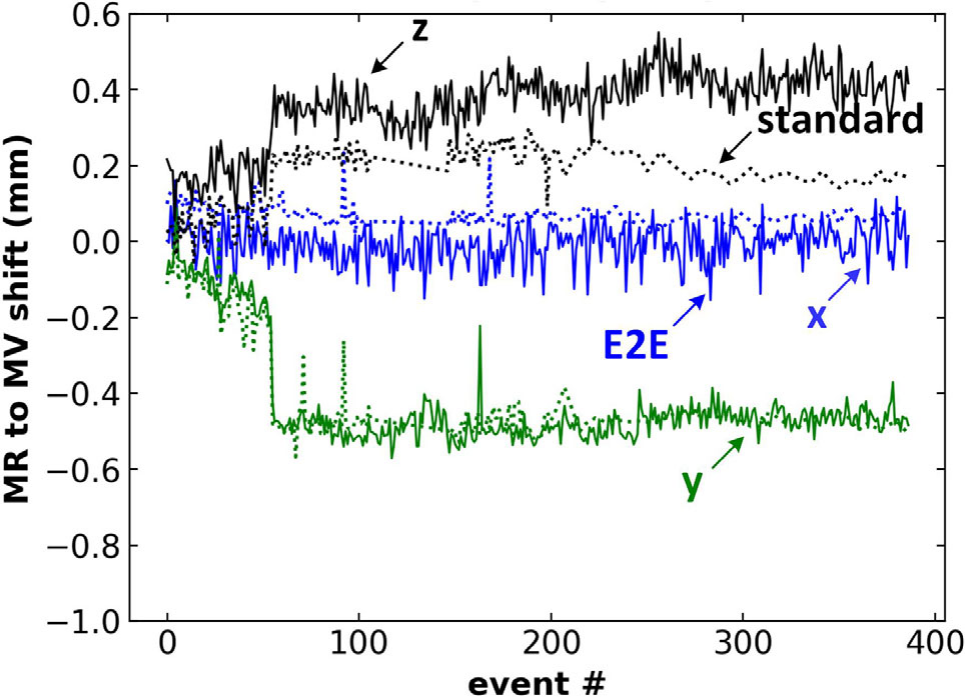
MR‐to‐MV shift in the x, y, and z directions as determined by the standard (dashed) daily Elekta test and the E2E (solid) automated QC test developed in this work.

In Figure 6, we plot the MR‐to‐MV alignment in the three cardinal directions as detected by the E2E test suite and by the standard Elekta‐provided testing software (v1.2.48.0). In this plot, we have date‐matched instances of the tests as we initially performed the standard Elekta MR‐to‐MV test daily and subsequently reduced the frequency to weekly. The distinct change, visible in both the standard Elekta and E2E test suite data, near the measurement 60 corresponds to a machine service event (electron gun physical repair) which resulted in a very slight but noticeable change in the beam spot position. Clinically, the MR‐to‐MV value was not adjusted at this time as the variation was within tolerance and brought the MR‐to‐MV alignment closer to the already set value. There is a distinct systematic difference, in the z direction, of roughly 0.2 mm between the Elekta and E2E test suite results. These differences are attributed to variations in detection algorithms and utilization of all seven fiducials versus the two which are used in our test suite. The tolerance for this test is set at ±0.5 mm with respect to the reference MR‐to‐MV value stored set in the Unity system.

## 4 DISCUSSION

Here we provide the design, validation, and initial results for a novel daily E2E test suite for the MR‐Linac system that quantifies all core components of the MR‐guided adaptive radiation therapy process. The need for this test exists due to the complex nature of the online adaptive process and the reliance on key machine performance, software performance, and data transfer components to successfully complete the adaptation. Chen et al., previously described a novel end‐to‐end test, called *ARTQA*, for both conventional and MR‐guided linacs but with differences in objectives and implementation.^6^, ^7^ Namely, the cited approach included qualitative assessment of MLC positioning and motion monitoring imaging, daily output, and secondary dose verification. Another E2E approach described by Subashi et al., using the Quasar MRI4D phantom, implemented an E2E test capable of verifying the 5‐field IMRT ATS or ATP generated Monaco plan dose against the delivered dose, measured via ion chamber and radiochromic film and additional system subcomponents were verified through supplementary tests.^10^ In our daily QA process daily output is performed using a standard ion chamber detector array, and our E2E test focuses on quantitative assessment of machine performance characteristics related to the geometric accuracy of the adaptive RT process. Our E2E test suite employs automatic fiducial detection on MR and MV imaging, as well as the jaw and MLC detection methods for the MV images of the adapted plans to quantify and assess long‐term trends. Our E2E test differs from alternative tests in the automation of processing of the adapted treatment plan, MR, and MV images. This approach produces an advantage in reproducibility and quantitates tracking of multiple machine parameters. Further, through automated processing, we can directly incorporate additional tests such as the MR‐to‐MV alignment and couch position reproducibility. Cai et al.^8^ have highlighted several potential failure modes in the MRL QA process and our E2E test address many of these in a single workflow. A recent report on QA recommendations for the Elekta Unity set tolerances for specific metrics and frequencies.^9^ With our automated QA approach, we can reduce the resource cost for performing QA, and thus increase frequency. For example, MR‐to‐MV alignment verification is recommended weekly with a 0.5 mm tolerance with respect to the baseline values; Leaf and diaphragm positions at a weekly frequency with a 1 mm tolerance; tabletop position monthly to 1 mm tolerance. Our implemented test covers these aspects along with a full workflow functionality evaluation. We have also demonstrated that our test provides the accuracy level required for the recommended tolerances.

A key component of the online adaptive process is the accurate localization of the phantom using image registration as well as the MR‐to‐MV alignment coincidence. With our test, we can determine the position of individual fiducials to within 0.08 ± 0.20 mm of manual localization. We demonstrate that we can track phantom shifts to within 0.26 mm (uncertainty convolved with phantom setup) and that we reproduce the stationary phantom position with a standard deviation of roughly 0.02 mm. Such accurate phantom position tracking in the MV and MR coordinates has allowed us to track the MR‐to‐MV alignment and detect differences at the level of 0.2 mm. We observe a systematic difference of around 0.2 mm in the x and y directions between our E2E test suite MR‐to‐MV results and those of the Elekta software. These are attributed to slight differences in algorithm approaches to fiducial localization. The inclusion of the MR‐to‐MV alignment as part of our E2E test suite, and the stability of the measurements, have allowed us to reduce the frequency of the Elekta MR‐to‐MV and reduce the daily machine startup process. We evaluate the performance of the image registration by detecting the phantom shift with respect to the reference position using the MV images. We found an increased frequency of data points >1 mm when comparing the image registration‐based and MV‐based shifts in the y‐direction. These fluctuations are on the order of the imaging slice thickness (1 mm) and are consistent with similar findings of Mittauer et al. for the ViewRay MRIdian MRL.^11^

Another important element of the test is the ability to accurately extract the position of the radiation edges. Given the field‐of‐view of the MV imaging panel, our test is limited in the magnitude of the adaptation and field size which can be interrogated. Previous work has demonstrated the performance and image quality of the MV panel in the MR environment.^12^ Our sensitivity analysis demonstrates that our detection algorithm is capable of tracking MLC deviations as large as 10 mm to within 0.55 mm and jaw deviation as large as 15 mm to within 1 mm (with most detections being within 0.5 mm). We've also demonstrated that the detection reproducibility of stationary MLCs is within 0.1 mm. We found that the introduction of the MR‐to‐MV phantom in the beam path results in at most –0.14 ± 0.13 mm deviation of the detected MLC edge. These tracking algorithms have permitted us to verify the delivery of the adapted fields with a 95% CI in the difference between the expected and detected positions of [–0.39 mm, 0.71 mm] for the jaws and [–0.53, –0.19 mm] for the MLCs. Combined this demonstrates the capability of our test to verify the MLCs and jaws to within recommended tolerance limits.^4^, ^9^ We have also expanded our test to be able to inform on individual MLC positions to provide further insight into machine performance.

We have shown tolerances that meet guidance criteria^4^ and demonstrated the capability of the test suite to capture the overall end‐to‐end workflow process prior to patient treatment. It is important to highlight test limitations to guide result interpretation. Due to the observed variation of the automatic fusion process in Online Monaco with the MR‐to‐MV phantom, the tolerance for detected versus planned phantom shift was set to ±1.5 mm. This limits the capability of our test suite to resolve if the correct MR‐to‐MV alignment is applied in Online Monaco if the alignment value is smaller than the tolerance. In these situations, a change less than the 1.5 mm alignment value tolerance would result in a shift of the phantom position comparison distribution and may not be explicitly flagged by the test suite. Further, our test suite does not directly evaluate the isocenter pixel value, which could be changed by a shift of the MV phantom or a change in the MV source. However, all MV position‐dependent tests would be impacted by a change to this parameter and would serve as an indication of the error source.

Adaptive MR‐guided RT QC requires a test that captures the core concepts of the daily end‐to‐end IGRT quality control test for CBCT‐based image guidance on conventional linear accelerators.^5^ For the CBCT‐based IGRT daily QC, a clinically relevant set of unique displacements in each coordinate axis are used to assess image registration, the couch shift conversion, the accuracy of remote‐controlled couch shifts, and overall end‐to‐end communication between subsystems.^13^ For adaptive RT on the Elekta Unity platform, MLC and jaw positions are altered at each fraction to account for the patient position variation at each fraction, so quantifying the accuracy of these adjustments using MV imaging was key to the translation of end‐to‐end IGRT QA to adaptive RT. Moreover, we included an MR‐to‐MV alignment check, which is analogous to collecting MV images following couch correction in the CBCT‐based IGRT QC. In our QC test, we focus on quantifying how beam segment aperture changes as a new treatment plan at each fraction based on the daily acquired image as opposed to couch shifts on a standard linac. We utilize the ATP workflow for our QC test due to the speed and reproducibility of beam apertures compared with an adapt‐to‐shape (ATS) workflow in which contour deformation and manual editing are performed, followed by a full inverse optimization to generate the adapted plan. In the future, it may be possible to implement an ATS version of this test using a deformable phantom, with the capability to generate consistent deformations, or by using an initially deformed image of a rigid phantom.

## 5 CONCLUSIONS

We have designed and validated an end‐to‐end QC test suite for the Elekta Unity MR‐linac. Our test suite can evaluate the entire ATP clinical workflow and verify image registration, adapted treatment delivery, couch position consistency, and MR‐to‐MV alignment. This test is performed in approximately 10–15 min and efficiently informs on specific machine performance elements as well as the functionality of the overall clinical workflow. We have implemented this test suite as a daily QC check prior to the first patient treatment and utilizing the Elekta‐provided MR‐to‐MV phantom provides the possibility of expansion of this test to other clinical installations.

## AUTHOR CONTRIBUTION

All authors have made substantial contributions to the work and development of this manuscript. All authors approved the manuscript.

## CONFLICT OF INTEREST

This work has been in part supported by Elekta.

## Supporting information



Supporting Information
