# Supplementary material for: MR‐linac daily semi‐automated end‐to‐end quality control verification
Source: J Appl Clin Med Phys. 2023 Feb 10;24(5):e13916. doi: 10.1002/acm2.13916 (PMC10161066; doi:10.1002/acm2.13916)
Supplement: Supplementary file 1 — Supporting Information [file ACM2-24-e13916-s001.docx]

# Supplemental Materials

## Detailed test sensitivity results

### Reproducibility

We setup the MR-to-MV phantom a single time and repeat the entire test suite 20 times without moving the phantom or the couch. This test removes the phantom position uncertainty and allows us to quantify the variability in the determined phantom shift by the E2E software and Online Monaco (through rigid registration resulting in an isocenter shift), the couch fiducial localization, and the MR-to-MV alignment.

We found that the couch position reproducibility had a 95% CI of [-0.28 mm, 0.24 mm] with respect to the mean. The 95% CI is close to the results obtain for all clinical test runs, indicating the uncertainty is mainly from fiducial detection. Online Monaco planned phantom shift was 9.32 ± 0.12 mm (x), 15.55 ± 0.08 mm (y), and 40.2 ± 0.3mm (z), while the MV detection-based measured phantom shift was 9.34 ± 0.02 mm (x), 15.15 ± 0.02 mm (y), and 40.18 ± 0.02 mm (z), and the difference in shifts had 95% CIs of [-0.28 mm, 0.16 mm] (x), [-0.47 mm, -0.23 mm] (y), and [-0.42 mm, 0.38 mm] (z). We note that the planned phantom shift has an increased standard deviation, likely related to the variation in the automatic image registration results. We found the MR-to-MV to be 0.08 [-0.15, -0.05] mm (x), -0.67 ± [-0.69, -0.65] mm (y), and 0.33 [0.28, 0.39] mm (z). Coupled with the low uncertainty of the MV-based phantom localization, these results indicate stability of the MR-based fiducial localization and registration. The difference between the planned and detected jaw and MLC edge positions was 0.17 mm [-0.44 mm, 0.68 mm] and -0.42 mm [-0.63 mm, -0.27 mm], respectively. We find these values consistent with the cumulative results reported in Section 3.2 as there is similar variability in jaw and MLC position definition, travel, and localization.

### Test-retest

For the repeatability assessment, we performed the E2E test 20 times. For each instance we re-setup the phantom and perform the full test with both no image registration and using the original plan segments or with rigid image registration and using adapted field segments (40 measurements total). This measurement allowed us to confirm that the adaptation in Online Monaco is correctly accounting for the phantom shift. In the E2E test, we determine the position of the phantom using only the MV images collected from the adapted segments since the fiducials are partially occluded by the MLCs and jaws when the original plan segments are used.

The comparison between couch position, jaw, and MLC positions, and the MR-to-MV values do not provide further insight into the test functionality. The difference between the detected and planned phantom shift was found to be 9.3 ± 0.3 mm (x), 15.2 ± 0.3 mm (y), and 40.12 ± 0.06 mm (z) when comparing to the un-adapted plans (no image registration), and 0.1 ± 0.3 mm (x), -0.2 ± 0.5 mm (y), and -0.1 ± 0.3 mm (z) when comparing to the fully adapted plans. We find these results consistent with the nominal shift introduced between the test phantom setup and the reference plan. Increased uncertainty in the adapted plan comparison is associated with variability in the automated image registration.

### Detection of errors in radiation field definition

For a single instance of the E2E test, we copy the adapted fields into the service mode of the Elekta Unity linac. We introduce shifts ranging from 1 to 15 mm to the jaw positions and 1 to 10 mm to the MLC positions (adjustments are made individually). We then collected MV images of the adjusted fields (one per gantry angle) three times. We used our E2E software to detect the jaw and MLC field edge and compared the detected shifts from the original field position against the introduced nominal shifts.

Over the range of 5 to -15 mm introduced jaw position errors, the E2E test suite software was able to detect the errors to within 0.5 mm for 250 of 265 measured jaw positions and within 1 mm for 15 measurements. We shifted the MLCs for gantry 0^o^ between -5 and +10 mm from the reference position, and we found that the E2E software detected these deviations to within 0.55 mm for all 30 MLC edge measurements.

To put the results above into context of MLC position reproducibility, we delivered an 18 cm x 8 cm field 20 times without having the MLC move between irradiations (the same field was left loaded) and without the phantom in the beam path. We allowed one minute between irradiations to reduce the impact of MV panel ghosting. We found that the average of all detected MLCs deviated by 0 ± 0.02 mm (1σ) from the mean of all the MLC measurements of individual field edges. We repeated this test and setup a different field in between each irradiation of the 18 cm x 8 cm fields. This results in the MLCs setting up anew between each irradiation. We observed the average of the MLC deviations to be 0 ± 0.07 mm (1σ) from the mean. Outliers (> ~0.1 mm deviation from the mean for individual MLCs) noticeably increased when allowing a different field to be set-up between irradiations. The maximum result was 0.35 mm from the mean while the maximum deviation for the stationary field irradiations was near 0.15 mm. These results indicate that the added uncertainty of MLC position variation (convolved with software detection uncertainty) is near 0.1 mm. Figure 1A provides box plots of individual MLC position variation for the measurements performed with and without introducing a field between irradiations.


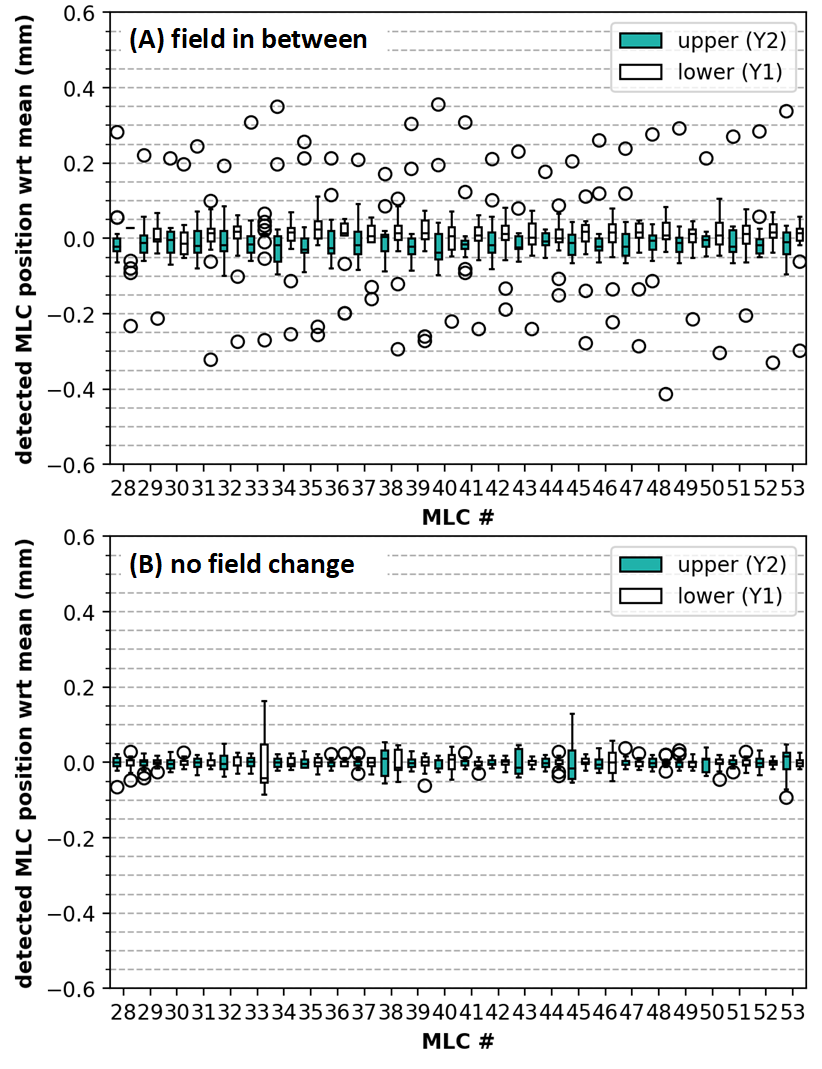


***Figure 1A:*** *Box plots of MLC position differences from mean of individual MLC positions when (A) introducing an intermediate field between irradiations and (B) maintaining the field definition between irradiations.*

### Influence of phantom on field edge detection

The presence of the MR-to-MV phantom can impact the localization of the radiation field edges due to image intensity irregularities. We reduce the impact of these irregularities by using a gradient-based edge detection algorithm instead of intensity-based. We evaluate the influence of the phantom on the localization of the field edge by defining, in the linac service mode, a set of fields which are of comparable geometry to the adapted fields produced during an E2E test instance. From each gantry angle, we deliver these fields 20 times with and without the phantom present. We do not define any other fields between each irradiation to eliminate MLC setup variability. We then compare the individual detected MLC positions with the phantom in the beam path with the case when the phantom is absent.

Figure 2A provides an error plot of the average difference for individual MLCs. For all the gantry angles the greatest magnitude average was -0.14 ± 0.13 mm for the lower (Y1) MLCs at gantry angle 270^o^. Most individual MLC results are within 0.2 mm with a few instances of average results near -0.4 mm. The upper (Y2) MLC position is less impacted by the presence of the phantom than the lower (Y1) MLCs.


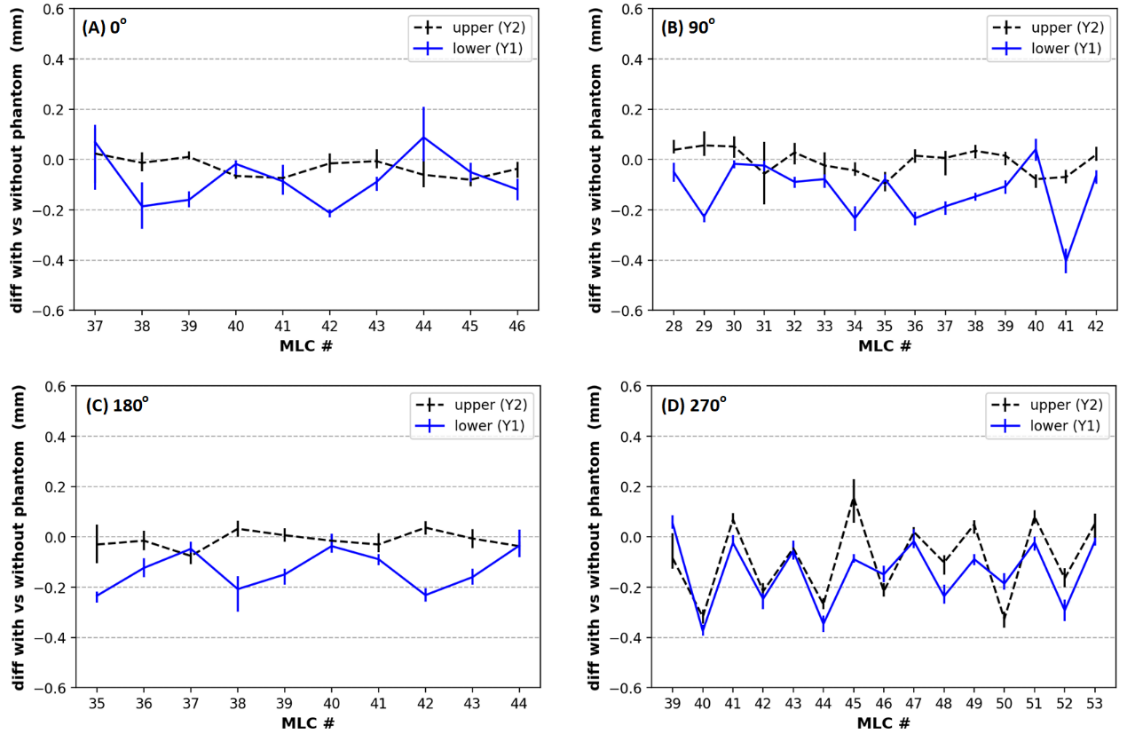


***Figure 2A:*** *Difference between detected MLC positions with and without the MR-to-MV phantom in the beam path. A single instance of adapted fields from the E2E test suite are used in these irradiations.*

### Detection of introduced phantom shifts

To evaluate the capability of the E2E test to detect phantom shifts in the MR and MV imaging coordinate systems, we designed a platform which allowed us to position the phantom rigidly on the Unity table. We then ran a single instance of the E2E test and saved the resulting adapted fields in the service mode of the linac. We introduced spacers to the platform to produce the required phantoms shifts in the three cardinal directions. The introduced shifts are either 1, 3, or 5 mm in each direction (independently). For each of the shifts and the reference position the MV and MR images are collected for processing using the E2E test suite software.

Table 1A shows the detected difference of the phantom position between the initial setup and the nominal shift positions. For each nominal shift (including the initial reference position), we setup the phantom three times and average the determined position over these three runs to produce the data in Table 1A. The uncertainty is approximately 0.26 mm, determined as a worst case of the standard deviation of all the phantom position setups. This variation stems mainly from the phantom setup uncertainty driven by the low uncertainties shown in MV and MR localization in section 3.2.1. Some crosstalk with the y-direction (within 0.6 mm) is observed for the z-direction nominal shifts and is attributed to the slightly more challenging setup required for producing the z-shifts.

| **Phantom shifts (x, y, z) [mm]** | | |
| --- | --- | --- |
| **Nominal** | **localized by MV** | **localized by MR** |
| (1, 0, 0) | (0.89, -0.05, -0.04) | (0.83, -0.06, -0.05) |
| (3, 0, 0) | (2.83, 0.11, -0.05) | (2.74, 0.07, -0.05) |
| (5, 0, 0) | (5.0, 0.31, -0.04) | (4.92, 0.29, -0.05) |
| (0, -1, 0) | (0.05, -0.76, -0.07) | (-0.02, -0.75, -0.06) |
| (0, -3, 0) | (0.18, -2.63, -0.05) | (0.08, -2.63, -0.07) |
| (0, -5, 0) | (0.08, -4.77, -0.04) | (0.03, -4.73, -0.07) |
| (0, 0, -1) | (-0.08, 0.04, -0.86) | (-0.13, 0.04, -0.94) |
| (0, 0, -3) | (-0.23, -0.61, -2.87) | (-0.27, -0.6, -2.91) |
| (0, 0, -5) | (-0.11, -0.4, -4.71) | (-0.12, -0.39, -4.85) |

Table 1A: Comparison of nominal, MV localized, and MR localized phantom shifts from the original position.

## Additional E2E test results

Figure 3A provides box plots of individual MLC positions calculated over the course of the presented E2E test suite instances. Figure 4A proivdes the historgrams of the differences between the planned and detected phantom position shift.


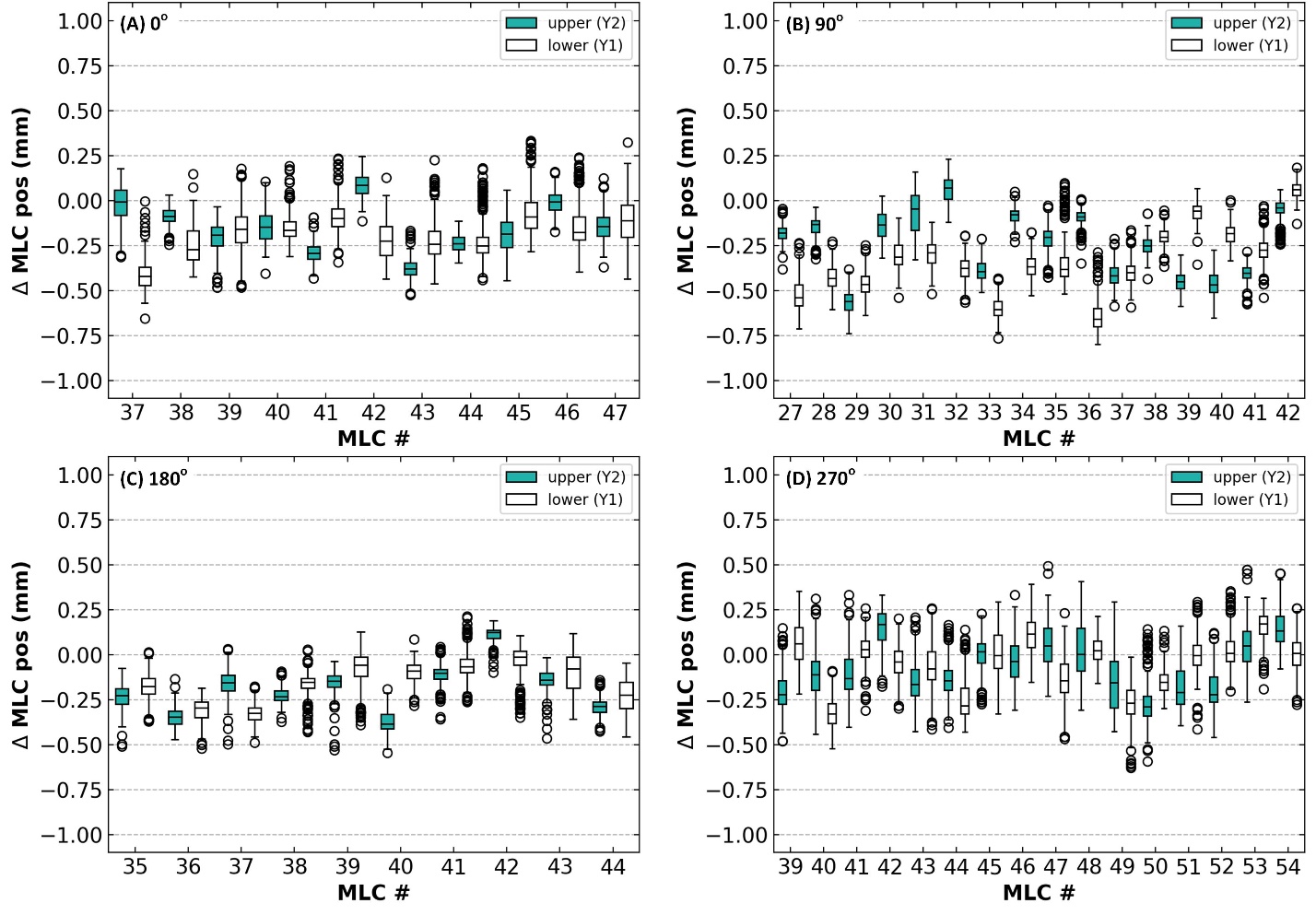


***Figure 3A:*** *Box plots of individual MLC position differences between planned and detected positions for all analyzed E2E test instances.*


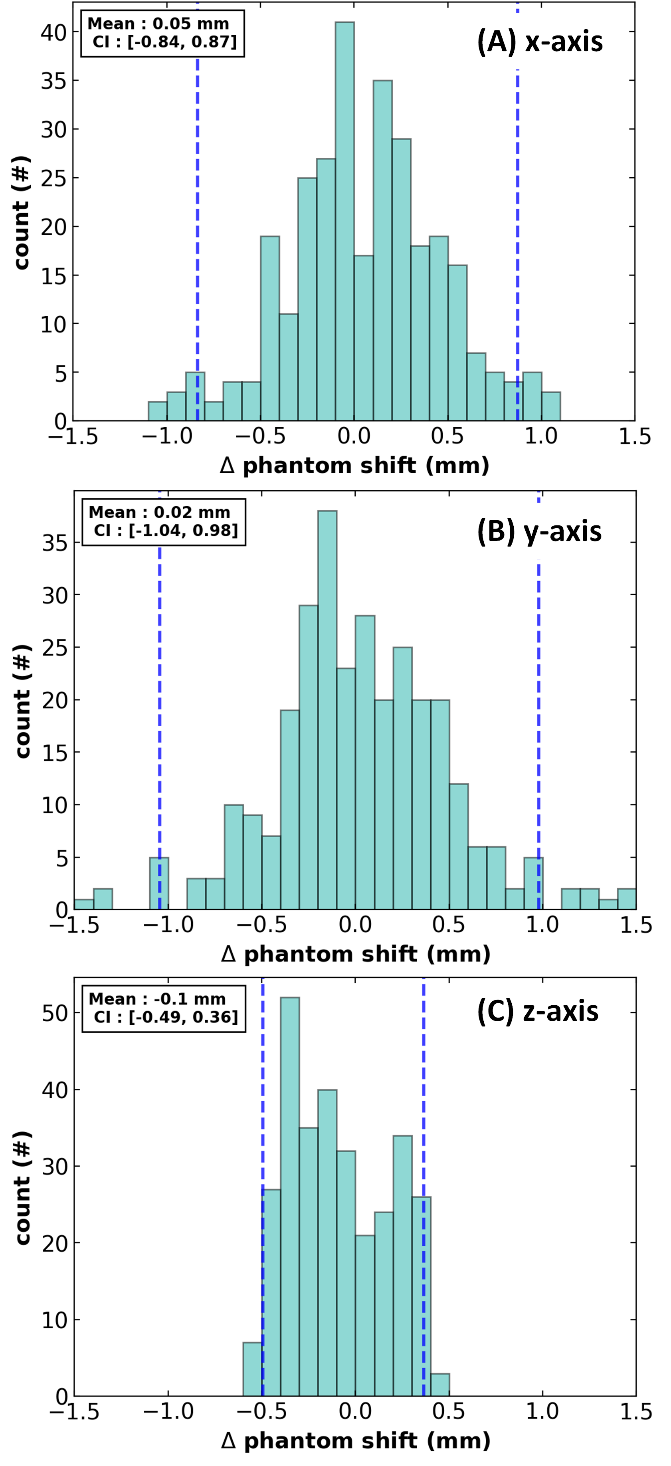


***Figure 4A.*** *Histograms of detected and plan phantom shift in the (A) x, (B) y, a*
